# Synthesis of Sr_6_LuAl(BO_3_)_6_:Sm^3+^ Red Phosphor with Excellent Thermal Stability and Its Application in w-LEDs

**DOI:** 10.3390/molecules29235495

**Published:** 2024-11-21

**Authors:** Anlin Zhang, Yue Yang, Yuqing Peng, Hao Zhou, Wei Tang, Jianhong Jiang, Yiting Wu, Shiying Cai, Lianwu Xie, Bin Deng

**Affiliations:** 1 College of Chemistry and Chemical Engineering, Central South University of Forestry and Technology, Changsha 410004, China; 2 School of Chemistry and Environmental Science, Xiangnan University, Chenzhou 423043, China; 3 Hunan Provincial Key Laboratory of Xiangnan Rare-Precious Metals Compounds Research and Application, Xiangnan University, Chenzhou 423043, China; 4 College of Chemistry & Pharmacy, Northwest A & F University, Yangling 712100, China

**Keywords:** borate, Sr_6_LuAl(BO_3_)_6_, Sm^3+^, luminescence, LEDs

## Abstract

In this study, a series of Sr_6_LuAl(BO_3_)_6_:Sm^3+^ red phosphors were successfully prepared with a high-temperature solid-phase technology. The Rietveld refinement analysis of the X-ray diffraction (XRD) diffraction patterns indicated that the as-prepared phosphors belong to the $R\bar{3}$ space group of the hexagonal crystal system. Under 404 nm near-ultraviolet excitation, the Sr_6_LuAl(BO_3_)_6_:Sm^3+^ phosphor exhibits narrowband emission within the range of 550 to 750 nm. The primary emission peak is observed at a wavelength of 599 nm, corresponding to ^6^H_5/2_ → ^4^F_7/2_. The optimum doping concentration of the Sr_6_LuAl(BO_3_)_6_:*x*Sm^3+^ phosphor is 10 mol%. Nearest-neighbor ion interaction is the mechanism of concentration quenching. The synthesized phosphors demonstrate exceptional thermal stability, with a high quenching temperature (*T*_0.5_ > 480 K). Furthermore, the assembled white light-emitting diode (w-LED) device exhibits a low color temperature (5464 K), an excellent color rendering index (*R*_a_ = 95.6), and CIE coordinates (0.333, 0.336) close to those of standard white light. Collectively, these results suggest the enormous potential of Sr_6_LuAl(BO_3_)_6_:Sm^3+^ phosphors for applications in w-LEDs.

## 1. Introduction

With advances in light source research, w-LEDs (white light-emitting diodes) have risen to prominence as innovative alternatives to traditional halogen and fluorescent lamps, marking a pivotal leap in lighting technology. Fluorescent conversion light-emitting diodes have become a hot research topic, driven by their compelling array of benefits such as high effectiveness, energy conservation, extended lifespan, safety, and environmental friendliness [1,2,3,4,5,6]. Commercially available w-LED phosphors typically consist of blue InGaN chips paired with yellow Y_3_Al_5_O_12_:Ce^3+^ phosphors. Another strategy is to prepare w-LEDs by coating monochromatic phosphors onto UV chips [7]. However, this approach has an inherent limitation: the lack of red emission, which leads to issues like elevated correlated color temperatures and suboptimal color rendering indices. To surmount these limitations, the incorporation of n-UV chips with a trichromatic combination of phosphors—green, red, and blue—has become recognized as the most efficacious strategy for obtaining w-LEDs [8,9,10,11,12]. The majority of general red phosphors are primarily composed of nitrides, such as Sr_2_Si_5_N_8_:Eu^2+^, Ba_2_Si_5_N_8_:Eu^2+^, and La_3_(Si,Al)_6_(O,N)_11_:Ce^3+^ phosphors [13,14,15]. Despite these advances, the synthesis of superior red phosphors is still quite a challenge, primarily due to the stringent requirements of high temperatures, pressures, and the need for a controlled atmosphere. Consequently, the development of new red phosphors that are easier to synthesize under more accessible conditions is urgently needed.

Rare earth (RE) ions play a pivotal role in the realm of display and lighting technologies, owing to their unique electronic transitions. Specifically, the *f-f* and *f-d* transitions of RE ions are responsible for producing narrowband and broadband emissions, respectively. Among these ions, Sm^3+^ stands out as a primary activator for generating red light in the visible spectrum. The doping of Sm^3+^ into matrix materials results in the acquisition of superior qualities of luminescence, which is due to the ^4^G_5/2_ → ^6^H*_J_* (*J* = 5/2, 7/2, 9/2, and 11/2) energy level transitions [16]. Luminous materials with doped Sm^3+^ ions, such as Sr_(3−2*x*)_Sm*_x_*Na*_x_*B_2_SiO_8_, Sr_3_Sc(PO_4_)_3_, and La(OH)₃ [17,18,19], are now available. 

Host materials with excellent properties are important for synthesizing high-quality phosphors. The standard chemical formula of orthoborates is A_6_MM’(BO_3_)_6_, where A = Sr, Ba, Pb, or lanthanide and M, M′ = +2, +3, or +4 metal cations [20]. Orthoborates have garnered a great deal of attention from scientific researchers due to their low synthesis temperature, high stability, and good thermal and physicochemical stability. Gao et al. found that by modifying the doping ratio of Eu^3+^ and Tb^3+^, the LiCaY_5_(BO_3_)_6_: Eu^3+^, Tb^3+^ phosphor can be made to exhibit tunable emission, spanning the color spectrum from green to yellow to red; this material is suitable for LED pumping [21]. According to Xu et al., Na-ion charge compensation brought about a notable enhancement in the blue emission intensity and quantum yield of Sr_6_GdSc(BO_3_)_6_:0.08Ce^3+^,0.08Na^+^ phosphors, which can be utilized for high-quality warm-white LED lighting [22]. Gao et al. investigated the high-pressure sensing properties of the LiCaY_5_(BO_3_)_6_:Ce^3+^, Tb^3+^ phosphors in optical pressure sensors [23].

In this study, a novel series of red Sr_6_LuAl(BO_3_)_6_:Sm^3+^ samples were successfully prepared by employing a high-temperature solid-phase method. Comprehensive characterizations were conducted, encompassing the phase purity, surface morphology, temperature-dependent phosphorescence spectra, and photoluminescence spectrum. Furthermore, we calculated the color rendering index (CRI), Commission International de L’Eclairage (CIE) chromaticity coordinates, and correlated color temperature (CCT) of the manufactured w-LED device to evaluate its optical performance. The electro-luminescence (EL) spectra of the fabricated w-LEDs were also thoroughly investigated.

## 2. Results and Discussion

**Figure 1:**
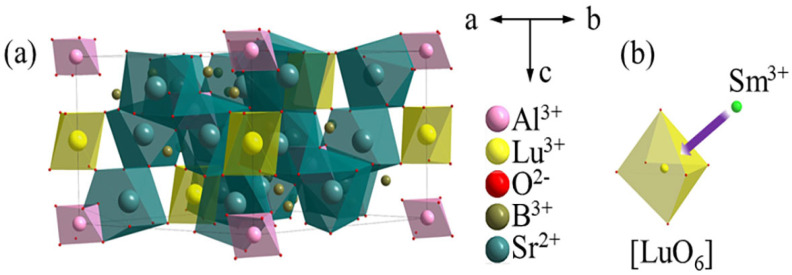
(**a**) Crystal structure of Sr_6_LuAl(BO_3_)_6_. (**b**) The octahedral structure of [LuO_6_].

Figure 1a,b illustrate the three-dimensional matrix lattice structure of Sr_6_LuAl(BO_3_)_6_. The Sr_6_LuAl(BO_3_)_6_ host belongs to the hexagonal crystal system with an $R\bar{3}$ space group. The lattice parameters of Sr_6_LuAl(BO_3_)_6_ are *a* = *b* = 12.1565 Å, *c* = 9.0853 Å, and *V* = 1162.7538 Å^3^. The Lu, Al, and Sr atoms are coordinated by six, six, and nine oxygen atoms to form the [LuO_6_] octahedron, [AlO_6_] octahedron, and [SrO_9_] polyhedron, respectively. Alternating layers of [AlO_6_] and [LuO_6_] octahedra are observed along the c-axis direction. The [AlO_6_] octahedron and [SrO_9_] polyhedron are interconnected via a shared triangular face. The [LuO_6_] octahedron and two [SrO_9_] polyhedra are interconnected via two oxygen atoms. B atoms fill the cavities formed by [AlO_6_] and [LuO_6_] octahedra and [SrO_9_] polyhedra.

**Figure 2:**
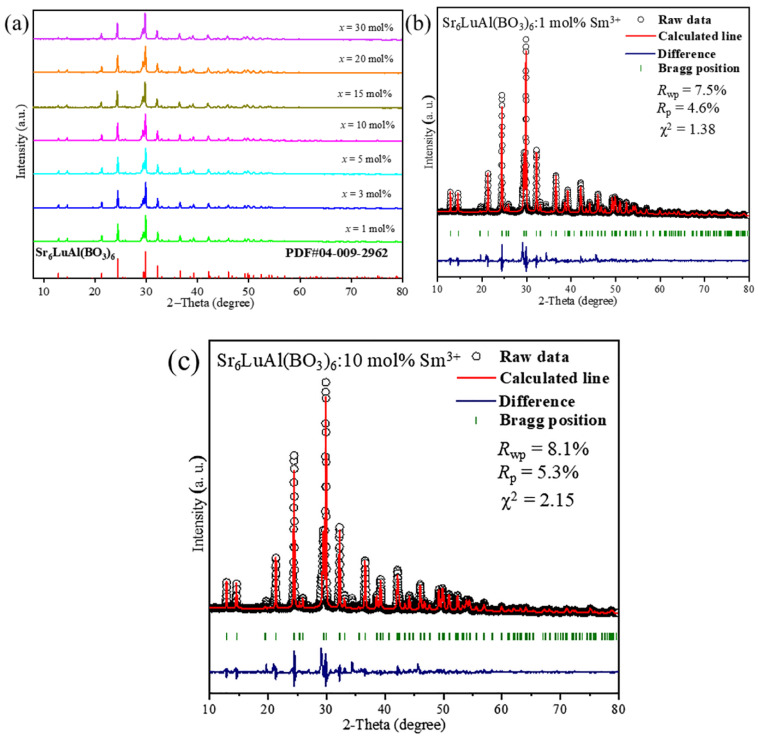
(**a**) XRD patterns of Sr_6_LuAl(BO_3_)_6_:*x*Sm^3+^ phosphors (1 ≤ *x* ≤ 30 mol%). (**b**,**c**) Rietveld refinement plots for 1 mol% and 10 mol% Sm^3+^-doped phosphors.

Figure 2a exhibits the XRD patterns of the prepared Sr_6_LuAl(BO_3_)_6_:*x*Sm^3+^ (1 ≤ *x* ≤ 30 mol%) phosphors. The diffraction patterns of the samples with different doping concentrations were well matched with the standard cards of the Sr_6_LuAl(BO_3_)_6_ matrix [20]. The purity of the as-synthesized phosphors was confirmed by the absence of impurity peaks in the XRD patterns. This indicates that the incorporation of Sm^3+^ ions did not induce the formation of any impurities, and no significant alterations were detected in the crystal structure. Since the atomic radii of Sm^3+^ (r = 0.958 Å) and Lu^3+^ (r = 0.861 Å) are close to each other [24,25], there is no discernible change in the locations of the diffraction peaks with variation in the Sm^3+^ doping concentration. The substitution of Sm^3+^ ions can be indicated by the radius percentage difference (*D*_r_). This can be computed through Equation (1) [26]:

(1) $$D_{r}=\frac{\left|R_{s}\left(CN\right)-R_{d}\left(CN\right)\right|}{R_{s}\left(CN\right)}\times 100\%$$

In this context, *D*_r_ denotes the differential radius percentage value, whereas *CN* represents the coordination number. The symbol *R*_s_ is used to represent the radius of the substituting ions, and *R*_d_ signifies the radius of the doped Sm^3+^ ions. The ionic radii of Sr^2+^, Lu^3+^, Al^3+^, and Sm^3+^ are 1.18, 0.861, 0.535, and 0.958 Å, respectively, when *CN* = 6. The *D*_r_ values are 18.81%, 11.27%, and 79.07% when the substituted ions are Sr^2+^, Lu^3+^, and Al^3+^, respectively. *D_r_* (Lu) has the lowest value, suggesting that Sm^3+^ ions are the most likely replacements for the Lu^3+^ sites.

Figure 2b,c illustrate the Rietveld refinements of 1 mol% and 10 mol% Sm^3+^-doped Sr_6_LuAl(BO_3_)_6_ samples by GSAS 3.0 software. Sr_6_LuAl(BO_3_)_6_ (PDF#04-009-2962) was used as the initial refinement model. The refinement factors for the two samples with different Sm^3+^ doping concentrations are *R*_wp_ = 7.5%, *R*_p_ = 4.6%, χ^2^ = 1.38 and *R*_wp_ = 8.1%, *R*_p_ = 5.3%, χ^2^ = 2.15, respectively, indicating that the refined outcomes are suitable. The refined crystal data are listed in Table 1.

**Figure 3:**
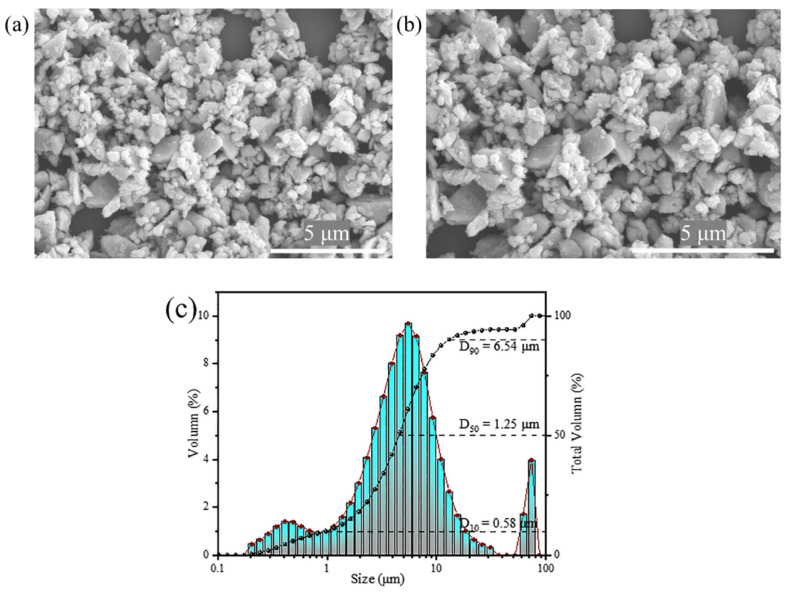
(**a**,**b**) SEM micrographs of Sr_6_LuAl(BO_3_)_6_:5 mol%Sm^3+^ at 8000× and 10,000× magnification. (**c**) The particle size distribution of the Sr_6_LuAl(BO_3_)_6_:10 mol%Sm^3+^ phosphor.

To investigate the morphological characteristics, the Sr_6_LuAl(BO_3_)_6_:5 mol%Sm^3+^ phosphor was observed by an SEM. Figure 3a,b show the SEM micrographs of the Sr_6_LuAl(BO_3_)_6_:5 mol%Sm^3+^ sample, showcasing the surface morphology at magnifications of 8000× and 10,000×, respectively. The microscopic analysis revealed that the sample exhibited irregular particles. The main particle size of the Sr_6_LuAl(BO_3_)_6_:10 mol%Sm^3+^ phosphor is 1.25 µm, as shown in Figure 3c. The results of the calculation of the parameters D_10_, D_50_, and D_90_ are 0.58, 1.25, and 6.54 μm. Accordingly, 90% of the particles have a diameter of less than 6.54 µm, 50% have a diameter of less than 1.25 µm, and 10% have a diameter of less than 0.58 µm. The granulometric composition is probably affected by the grinding time, the mortar material, and other factors. To increase the quality of the powders and break up the agglomerations, additional ball-milling and screening procedures are required. These results suggest that the synthesized phosphor possesses properties that render it highly suitable for application in w-LEDs.

**Figure 4:**
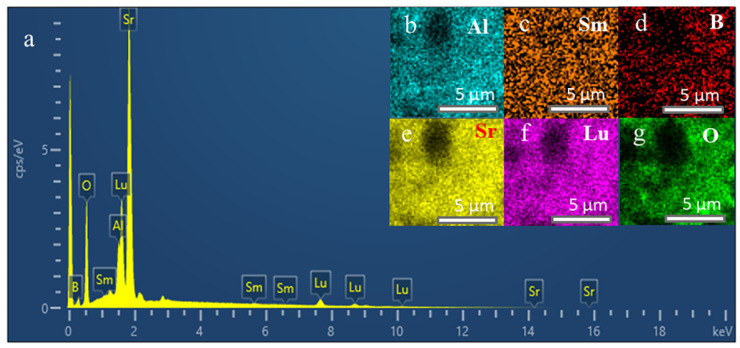
(**a**) The EDS results for the Sr_6_LuAl(BO_3_)_6_:5 mol%Sm^3+^ sample. (**b**–**g**) Elemental mappings of Al, Sm, B, Sr, Lu, and O.

Figure 4 shows the elemental distribution of the Sr_6_LuAl(BO_3_)_6_:5 mol%Sm^3+^ phosphor. The existence of Al, Sm, B, Sr, Lu, and O elements in the obtained Sr_6_LuAl(BO_3_)_6_:5 mol%Sm^3+^ sample is exhibited in Figure 4a. Figure 4b–g show that the Al, Sm, B, Sr, Lu, and O elements are uniformly distributed within the selected particle. These results further demonstrate that Sr_6_LuAl(BO_3_)_6_:Sm^3+^ phosphors were successfully synthesized.

**Figure 5:**
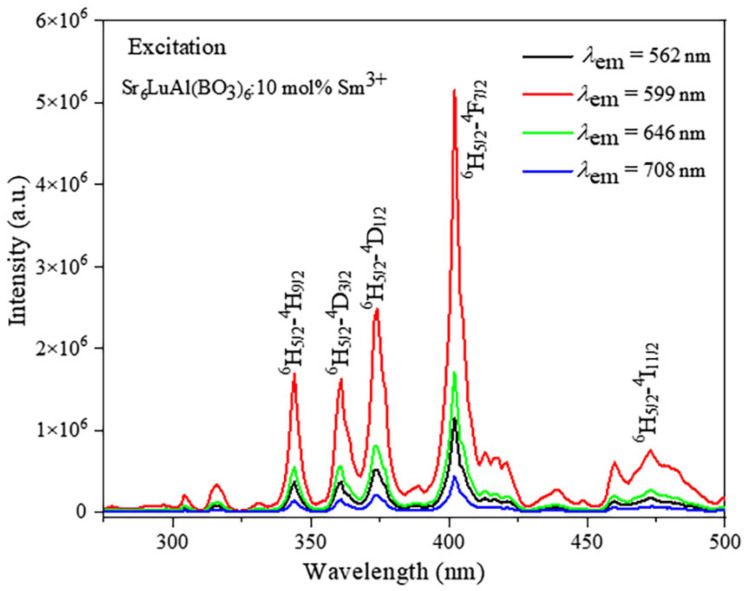
Excitation spectra of Sr_6_LuAl(BO_3_)_6_:10 mol%Sm^3+^.

Figure 5 exhibits the PLE spectra of the Sr_6_LuAl(BO_3_)_6_:10 mol%Sm^3+^ sample monitored at 562, 599, 646, and 708 nm. Though their intensities differ, the shape and position of all absorption peaks are nearly identical. The excitation spectra of Sm^3+^ ions stem from their intra-configurational 4*f-*4*f* transitions, manifesting as distinct peaks at 346, 363, 376, 404, and 475 nm, corresponding to (^6^H_5/2_ → ^4^H_9/2_), (^6^H_5/2_ → ^4^D_3/2_), (^6^H_5/2_ → ^4^D_1/2_), (^6^H_5/2_ → ^4^F_7/2_), and (^6^H_5/2_ → ^4^I_11/2_), respectively [27]. Among the observed excitation peaks, the peak at 404 nm is the most prominent, which is attributed to ^6^H_5/2_ → ^4^F_7/2_ energy transitions.

**Figure 6:**
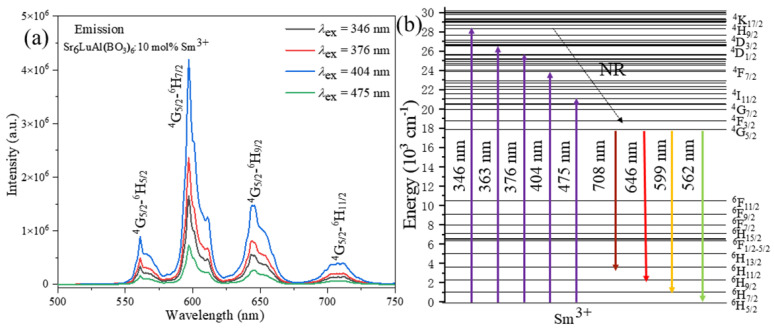
(**a**) PL spectra of Sr_6_LuAl(BO_3_)_6_:10 mol%Sm^3+^. (**b**) Luminescence mechanism of Sm^3+^.

The emission spectra for the Sr_6_LuAl(BO_3_)_6_:10 mol%Sm^3+^ sample under 346, 376, 404, and 475 nm excitation are shown in Figure 6a. Under excitation at different wavelengths, the Sr_6_LuAl(BO_3_)_6_:10 mol%Sm^3+^ sample exhibits four prominent emission peaks. These peaks are centered at 562, 599, 646, and 708 nm, corresponding to the ^4^G_5/2_ → ^6^H_5/2_, ^4^G_5/2_ → ^6^H_7/2_, ^4^G_5/2_ → ^6^H_9/2_, and ^4^G_5/2_ → ^6^H_11/2_ electronic transitions of the Sm^3+^ ions, respectively [18]. The peak at 599 nm presents the strongest emission. The energy level chart for Sr_6_LuAl(BO_3_)_6_:Sm^3+^ is presented in Figure 6b. It effectively displays the luminescence mechanism of Sm^3+^ in the Sr_6_LuAl(BO_3_)_6_ host lattice. Under 404 nm excitation, electrons in the Sr_6_LuAl(BO_3_)_6_:Sm^3+^ phosphor absorb energy and are promoted from the ground state to the excited state. Subsequently, they undergo a non-radiative transition (NR) to the ^4^G_5/2_ energy level, which is the lowest excited state. Ultimately, through radiative transitions, the electrons relax to the ground state, emitting light at wavelengths of 562, 599, 646, and 708 nm. These emissions correspond to ^6^H*_J_* (*J* = 5/2, 7/2, 9/2, and 11/2).

It is widely recognized that the electric dipole (ED) and magnetic dipole (MD) transitions are associated with the ^4^G_5/2_ → ^6^H_9/2_ and ^4^G_5/2_ → ^6^H_5/2_ transitions of Sm^3+^ ions, respectively. The symmetry of the Sm^3+^ environment within the matrix lattice can be inferred from the intensity ratio of ED to MD transitions. Specifically, in this case, compared to the MD transition at 562 nm, the ED transition at 646 nm is more intense, indicating that the Sm^3+^ ions occupy sites with low symmetry within the Sr_6_LuAl(BO_3_)_6_ matrix lattice [28].

**Figure 7:**
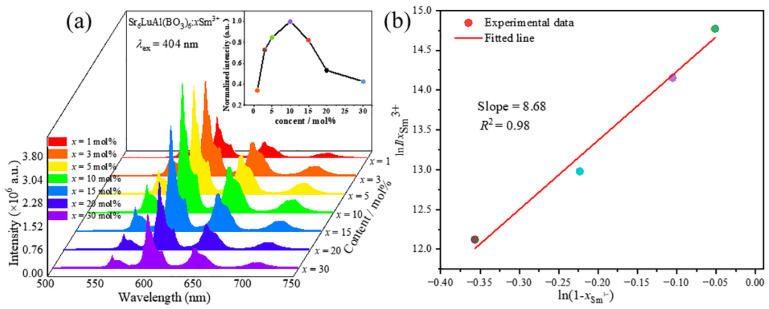
(**a**) The correlation between the Sm^3+^ doping concentration and luminescence intensity. (**b**) The $\ln\left(I/x_{Sm^{3+}}\right)$~$\ln\left(1-x_{Sm^{3+}}\right)$ fitted curve.

The correlation between the concentration of Sm^3+^ ions and the luminous intensity of Sr_6_LuAl(BO_3_)_6_:Sm^3+^ phosphors is significant. Determining the optimum doping concentration is necessary. The relationship between the doping concentration of Sm^3+^ ions and the luminescence intensity is shown in Figure 7a. The position and shape of the emission peaks remain unaffected with an increasing concentration of Sm^3+^ ions. The emission intensity increases gradually as the Sm^3+^ ion concentration increases, achieving a maximum at a concentration of 10 mol%. Beyond this concentration, concentration quenching results in a gradual decrease in the luminescence intensity.

In line with L. Ozawa’s hypothesis [29], the following formula is usually used to determine the doping concentration (*x*) and luminescence intensity (*I*) (2):

(2) $$I=Bx{\left(1-x\right)}^{Z}$$

Here, *Z* indicates the number of metal cation species, and *B* represents a constant. Subsequently, the term $1/\left(1+Z\right)$ signifies the concentration at which quenching occurs. Consequently, by transforming the equation, the following formula can be derived (3) [30]:

(3) $$\ln\left(\frac{I}{x}\right)=Z\ln\left(1-x\right)+C$$

Figure 7b shows the fitting curve of $\ln\left(I/x_{Sm^{3+}}\right)$~$\ln\left(1-x_{Sm^{3+}}\right)$. By employing the doping concentration in the line fitting, a linear connection is established. The calculated slope yields a *Z* value of 8.68. Consequently, the calculated quenching concentration is $1/\left(1+Z\right)=0.103$, which is extremely close to the experimentally measured optimal doping concentration of 10 mol%.

Two mechanisms have been identified as being responsible for concentration quenching: electrical multipolar interactions and exchange interactions, which are discriminated by the critical distance *R*_c_. The equation formulated by Blasse and Grabmaier serves to calculate the *R*_c_ value and is expressed as follows (4) [31]:

(4) $$R_{c}\approx 2{\left(\frac{3V}{4\pi x_{c}Z}\right)}^{\frac{1}{3}}$$

Here, *V* denotes the crystalline cell volume, *x*_c_ signifies the critical concentration, and *Z* represents the number of metal cations per crystalline cell. In this study, *V* = 1162.7538 Å^3^, *x*_c_ = 10 mol%, and *Z* = 4; subsequently, 8.85 Å is determined as the value of *R*_c_. If the critical distance *R*_c_ is <5 Å, the process is indicative of energy transfer through exchange interactions. Conversely, if *R*_c_ > 5 Å, the interaction is characterized as multipolar. The calculations confirm that concentration quenching is predominantly attributed to multipolar interactions, as evidenced by a critical distance *R*_c_ of >5 Å.

**Figure 8:**
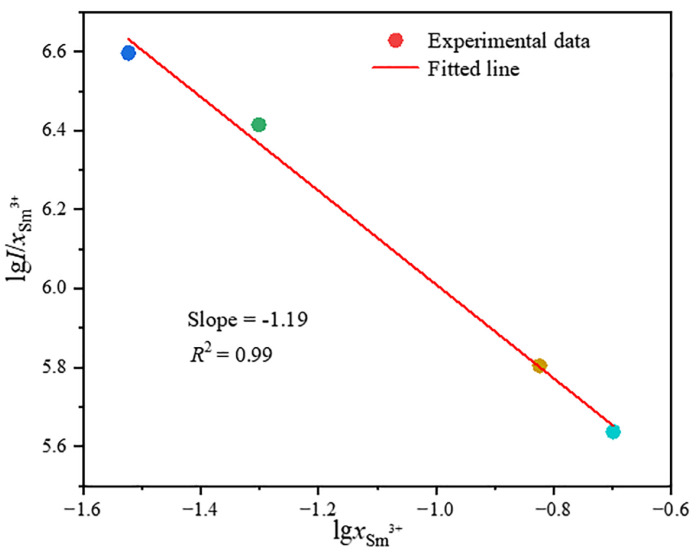
The ${\mathrm{lg}}_{x_{Sm^{3+}}}$~$\mathrm{lg}\left(I/x_{Sm^{3+}}\right)$ curve for Sr_6_LuAl(BO_3_)_6_:*x*Sm^3+^.

The concentration quenching mechanism of Sr_6_LuAl(BO_3_)_6_:*x*Sm^3+^ phosphors can be further explored by utilizing the following equation, which verifies the concentration quenching process (5) [32]:

(5) $$\frac{I}{x}=\frac{K}{1+\beta {\left(x\right)}^{\frac{Q}{3}}}$$

where *x* represents the Sm^3+^ concentration; *I* stands for the luminescence intensity at various concentrations of Sm^3+^; *β* and *K* are both constants; and *Q* determines the type of multipolar interaction. Concretely, *Q* provides the values 3, 6, 8, and 10, which correspond to nearest-neighbor ion, dipole–dipole, dipole–quadrupole, and quadrupole–quadrupole interactions, respectively [32]. The variations in ${\mathrm{lg}}_{x_{Sm^{3+}}}$ and $\mathrm{lg}\left(I/x_{Sm^{3+}}\right)$ at 404 nm excitation are presented in Figure 8. It is possible to fit every data point accurately as a linear function. Therefore, $-\frac{Q}{3}=-1.19$ and, thus, *Q* = 3.57, demonstrating that nearest-neighbor ion interaction is the primary method of energy transmission between Sm^3+^ ions.

**Figure 9:**
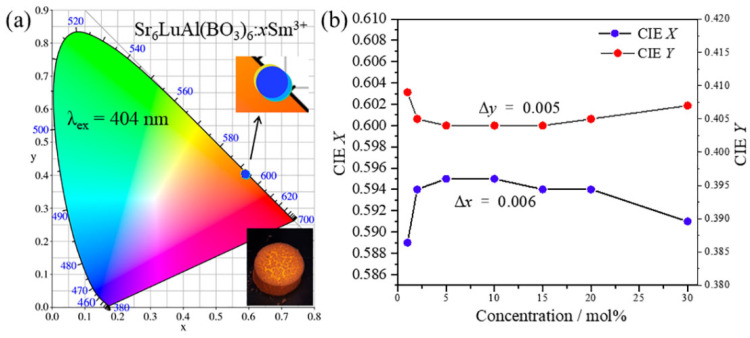
(**a**) Chromaticity coordinates of Sr_6_LuAl(BO_3_)_6_:*x*Sm^3+^ samples (inset: partial enlargement). (**b**) CIE variation for the Sr_6_LuAl(BO_3_)_6_:*x*Sm^3+^ samples.

Figure 9a illustrates the CIE chromaticity coordinates of the Sr_6_LuAl(BO_3_)_6_:*x*Sm^3+^ samples. The inset presents a partial enlargement. Obviously, all CIE chromaticity coordinates are within the red zone and remain essentially unchanged. The CIE chromaticity coordinates of Sr_6_LuAl(BO_3_)_6_:*x*Sm^3+^ are (0.589, 0.409), (0.594, 0.405), (0.595, 0.404), (0.595, 0.404), (0.594, 0.404), (0.594, 0.405), and (0.591, 0.407). Figure 9b exhibits the CIE variations for the Sr_6_LuAl(BO_3_)_6_:*x*Sm^3+^ phosphors. The CIE chromaticity coordinates exhibit variation with changes in the Sm^3+^ ion concentration. The differences between the maximum and minimum values are ∆*x* = 0.006 and ∆*y* = 0.005. Such minimal variance in the CIE chromaticity coordinates indicates that the Sr_6_LuAl(BO_3_)_6_:*x*Sm^3+^ phosphors possess excellent color stability.

Equation (6) can be used to calculate the color purity of Sr_6_LuAl(BO_3_)_6_:*x*Sm^3+^ phosphors [33]:

(6) $$Color\;purity=\frac{\sqrt{{\left(x-x_{i}\right)}^{2}+{\left(y-y_{i}\right)}^{2}}}{\sqrt{{\left(x_{d}-x_{i}\right)}^{2}+{\left(y_{d}-y_{i}\right)}^{2}}}$$

where (*x*, *y*) represents the CIE chromaticity coordinates of the obtained sample, and $\left(x_{d},y_{d}\right)$ and $\left(x_{i},y_{i}\right)$ are the chromaticity coordinates of the standard white illuminant (0.333, 0.333) and dominant wavelength point, respectively. The CIE chromaticity coordinates and color purity of Sr_6_LuAl(BO_3_)_6_:*x*Sm^3+^ phosphors at various concentrations are listed in Table 2. The color purity of the studied samples remained almost unchanged, which indicates that the Sr_6_LuAl(BO_3_)_6_:*x*Sm^3+^ samples have excellent color purity.

Equation (7) [34] was used to calculate the CCT of the Sr_6_LuAl(BO_3_)_6_:*x*Sm^3+^ phosphors (*x* = 1–30 mol%):

(7) $$CCT=-449n^{3}+3625n^{2}-6823.3n+5520.33$$

where *n* equals $\left(x-x_{e}\right)/\left(y-y_{e}\right)$ $\left(x_{e}=0.332,y_{e}=0.186\right)$. By means of the formula, the calculated CCTs are 1159, 1513, 1507, 1502, 1513, 1518, and 1540 K. All the calculated data are listed in Table 2. Incorporating the findings from previous studies indicates that all of the prepared Sr_6_LuAl(BO_3_)_6_:*x*Sm^3+^ phosphors exhibit an exceptionally high degree of color purity, exceeding 99.6%, along with a desirable low CCT. In light of the aforementioned analysis, the Sr_6_LuAl(BO_3_)_6_:*x*Sm^3+^ phosphor demonstrates promising characteristics as a prospective red-emitting material for w-LED applications.

**Figure 10:**
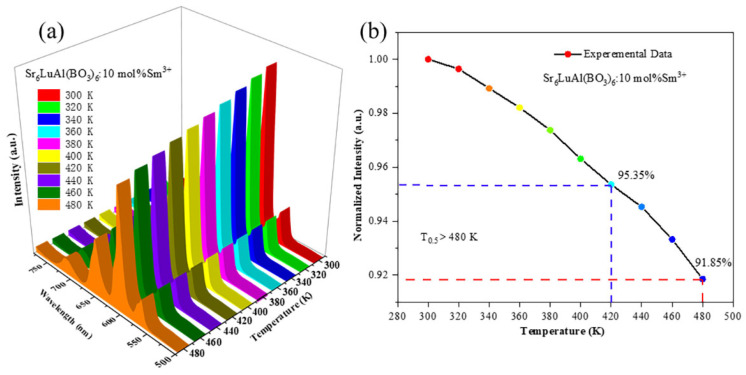
(**a**) Temperature-dependent photoluminescence spectra of the Sr_6_LuAl(BO_3_)_6_:10 mol%Sm^3+^ phosphor (*λ*_ex_ = 404 nm). (**b**) Relationship between temperature and emission intensity.

Thermal stability is a crucial performance metric for phosphors employed in w-LED applications. The temperature-dependent luminescence spectra of the Sr_6_LuAl(BO_3_)_6_:10 mol%Sm^3+^ phosphor are presented in Figure 10a, which shows a steady decline in luminescence intensity as the temperature increases. Notably, as the temperature rises from 300 K to 480 K, the spectral shape and the position of the emission peak remain unchanged, indicating that the phosphor exhibits exceptional heat resistance. Figure 10b depicts the emission intensity as a function of temperature. The temperature *T*_0.5_ represents the point at which the emission intensity drops to 50% of the emission intensity at ambient temperature. At 420 K, the Sr_6_LuAl(BO_3_)_6_:10 mol% Sm^3+^ phosphor retains 95.35% of its original intensity. Remarkably, even at an elevated temperature of 480 K, the luminous intensity is still 91.85% of the initial value, surpassing the halfway mark. The phosphor’s *T*_0.5_ is significantly higher than 480 K, underscoring its outstanding thermostability [35]. When compared to Sm^3+^-doped phosphors reported in the recent literature, as summarized in Table 3, the Sr_6_LuAl(BO_3_)_6_:Sm^3+^ phosphor exhibits a comparatively higher *T*_0.5_ value. Consequently, the red-emitting Sr_6_LuAl(BO_3_)_6_:Sm^3+^ phosphors are deemed highly promising for w-LED applications.

**Figure 11:**
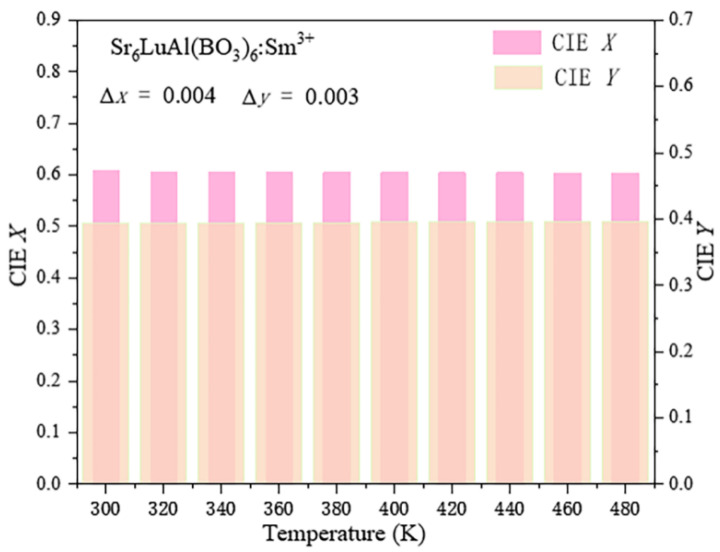
Variation in CIE chromaticity coordinates of Sr_6_LuAl(BO_3_)_6_:10 mol%Sm^3+^ phosphor with temperature.

Figure 11 displays the CIE chromaticity coordinates for the Sr_6_LuAl(BO_3_)_6_:10 mol%Sm^3+^ phosphor across varying temperatures. Obviously, the observation that the *x* and *y* values change only slightly suggests that the CIE chromaticity coordinates of the manufactured phosphors remain highly stable, indicating consistent color purity across a range of temperatures. The maximum variations in the CIE chromaticity coordinates were found to be ∆*x* = 0.004 and ∆*y* = 0.003. This illustrates that the obtained Sr_6_LuAl(BO_3_)_6_:10 mol%Sm^3+^ phosphor has excellent color stability.

**Figure 12:**
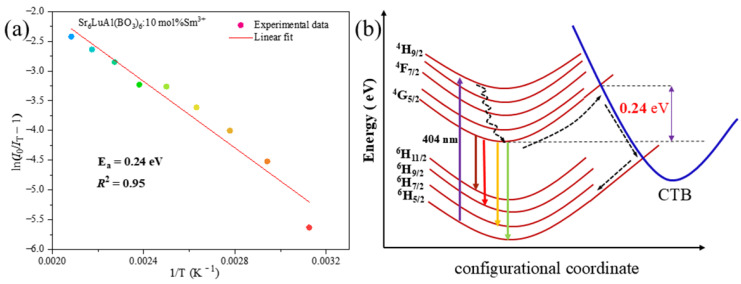
(**a**) Plot of $\ln\left[\left(I_{0}/I\right)-1\right]$ versus 1T for Sr_6_LuAl(BO_3_)_6_:*x*Sm^3+^. (**b**) Configurational coordinate diagram of Sm^3+^.

To elucidate the thermal quenching mechanism of the Sr_6_LuAl(BO_3_)_6_:Sm^3+^ phosphor, an in-depth investigation was conducted. The activation energy (*E*_a_) is a crucial parameter that can be determined using the Arrhenius Equation (8) [41]:

(8) $$I_{T}=\frac{I_{0}}{1+cexp^{\left(\frac{-E_{a}}{kT}\right)}}$$

where *k* and *c* are constants, and $I_{0}$ and $I_{T}$ represent the emission intensities at ambient temperature and temperature *T* (K), respectively. An estimate of *E*_a_ can be obtained by plotting $\ln\left[\left(I_{0}/I\right)-1\right]$ versus 1T. As exhibited in Figure 12a, the calculated *E*_a_ value of the prepared Sm^3+^ sample is 0.24 eV. This outcome is better than those for several red phosphors doped with Sm^3+^, such as CaSr_2_(PO_4_)_2_ (0.127 eV), Ca_6_BaP_4_O_17_ (0.118 eV), and Ca_5_(PO_4_)_2_SiO_4_ (0.13 eV) [42,43,44], which illustrates that the synthesized phosphor exhibits favorable thermal stability properties. The electron transition mechanism of the fabricated phosphor is illustrated in Figure 12b. Electrons initially residing in the ^6^H_5/2_ ground state are excited to the ^4^H_9/2_ energy level upon 404 nm photon absorption. Subsequently, they undergo a non-radiative transition to a lower excited state before relaxing to the ^4^G_5/2_ level. A portion of these electrons then transitions from the ^4^G_5/2_ excited state back to the ground state via a radiative process, emitting light in the process. Some electrons, influenced by a rise in temperature, absorb thermal energy sufficient to surpass the activation energy barrier *E*_a_. This allows them to leap across the energy gap from the ^4^G_5/2_ level to CTB, which can have unrestricted movement. These electrons subsequently undergo a non-radiative transition to the ^6^H_5/2_ energy level. As the temperature rises, this subset of electrons becomes increasingly active, leading to a decrease in the luminescence intensity of the Sm^3+^-doped phosphor. This phenomenon is attributed to thermal quenching.

**Figure 13:**
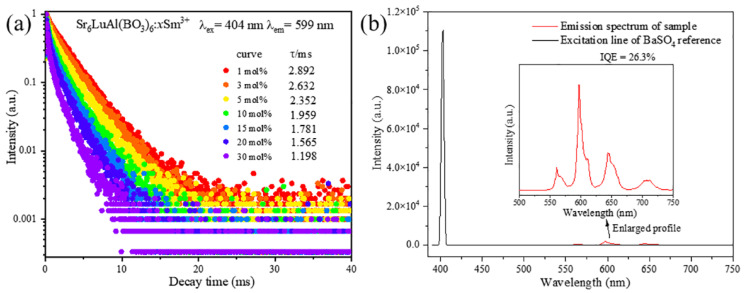
(**a**) Luminescence decay curves of Sr_6_LuAl(BO_3_)_6_:*x*Sm^3+^ phosphors (1 ≤ *x* ≤ 30 mol%). (**b**) The excitation line of the BaSO_4_ reference and the emission spectrum of the Sr_6_LuAl(BO_3_)_6_:10 mol%Sm^3+^ phosphor collected using an integrating sphere. Inset: An enlarged profile of the emission spectrum from 500 nm to 750 nm.

Under 404 nm excitation, the Sr_6_LuAl(BO_3_)_6_:*x*Sm^3+^ (1 ≤ *x* ≤ 30 mol%) phosphors’ luminescence decay curves are shown in Figure 13a. According to the decay theory, the lifetime of the phosphors was calculated by the following formula (9) [45]:

(9) $$I_{\left(t\right)}=I_{0}+A_{1}exp(-\frac{t}{\tau_{1}})+A_{2}exp(-\frac{t}{\tau_{2}})$$

where *A*_1_ and *A*_2_ are fitting parameters, *I*_(*t*)_ is the luminescence intensity at time *t*, and $\tau_{1}$ and $\tau_{2}$ represent the lifetimes of the slow and fast decay processes, respectively. The average lifetime of the phosphors was calculated using Formula (10) [46]:

(10) $$\tau_{\mathrm{avg}}=\frac{A_{1}\tau_{1}^{2}+A_{2}\tau_{2}^{2}}{A_{1}\tau_{1}+A_{2}\tau_{2}}$$

The average lifetimes of the Sr_6_LuAl(BO_3_)_6_:*x*Sm^3+^ phosphors (1 ≤ *x* ≤ 30 mol%) were calculated to be 2.892, 2.632, 2.35, 1.959, 1.781, 1.565, and 1.198 ms. These results show that the decay time decreases with an increase in the Sm^3+^ ion concentration. This is attributed to more frequent energy transfer between Sm^3+^ ions, which leads to an increase in non-radiative transition; therefore, the luminescence lifetime of the Sr_6_LuAl(BO_3_)_6_:*x*Sm^3+^ phosphor decreases [47]. IQE is an important metric for phosphors applied in w-LED devices. Figure 13b shows that the quantum yield of the Sr_6_LuAl(BO_3_)_6_:10 mol%Sm^3+^ phosphor is 26.3%, indicating that it is suitable for w-LEDs.

**Figure 14:**
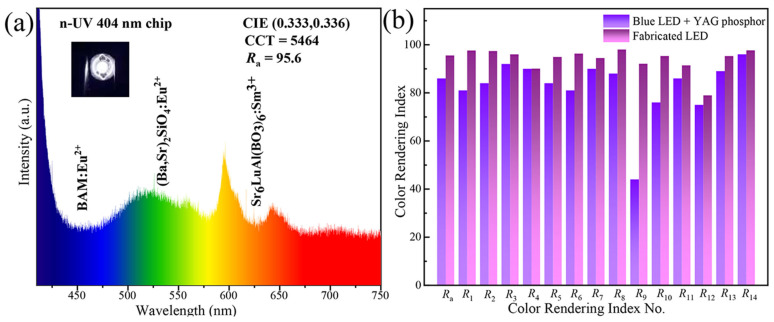
(**a**) The EL spectrum of the prepared w-LED. (Inset: An image of the w-LED). (**b**) A color rendering index comparison of commercial and manufactured w-LEDs from *R*_1_ to *R*_14_.

The EL spectrum of the prepared w-LED is depicted in Figure 14a. The fabricated w-LED integrates a commercial blue BaMgAl_10_O_17_:Eu^2+^ (BAM:Eu^2+^) phosphor and a green (Ba, Sr)_2_SiO_4_:Eu^2+^phosphor, along with the newly prepared red-emitting Sr_6_LuAl(BO_3_)_6_:Sm^3+^ phosphor. The weight ratio of blue phosphors, green phosphors, and red phosphors is 1:1:10. This trichromatic combination is excited by a 404 nm n-UV chip, causing the white LED to emit bright white light. An inset depicts the manufactured w-LED radiating intense white light. The CIE chromaticity coordinates were precisely determined at (0.333, 0.336), indicating a well-balanced color rendition. The manufactured w-LED boasts a superior color rendering index (*R*_a_) of 95.6, surpassing that of commercially available w-LEDs. The performance metrics of the developed w-LED are comparatively favorable when juxtaposed with the phosphors reported in the recent literature, as detailed in Table 4. The color rendering indices from *R*_1_ to *R*_14_ are exhibited in Figure 14b. *R*_a_ is an important metric for w-LEDs and includes a number of parameters, where *R*_a_ denotes the mean value of the color rendering indices for *R*_1_ through *R*_8_, and *R*_1_-*R*_14_ represent various types of colors. *R*_9_ represents saturated red. The *R*_9_ value (92) of the prepared w-LED is much greater than that of a commercial w-LED (43). The optimized w-LED device shows a bright white light with a high luminous efficacy of up to 17.83 lmW^−1^. Therefore, it is expected that the synthesized phosphors will be applied to w-LEDs.

**Figure 15:**
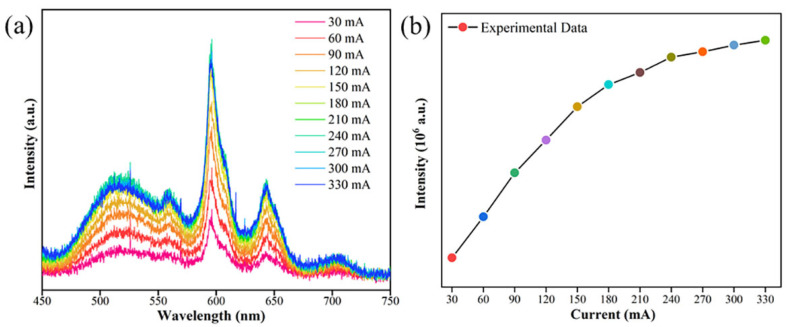
(**a**) EL curves of the manufactured white LED at various currents (60–330 mA). (**b**) Luminescence intensity versus current variation curve.

The variation in the electroluminescence spectra under different current intensities is illustrated in Figure 15a. As the current is progressively increased from 30 to 330 mA, the overall shape of the EL spectra and the position of the emission peak are observed to remain substantially constant. Figure 15b illustrates a marked escalation in luminescence intensity concomitant with an increase in current. These observations indicate that the Sr_6_LuAl(BO_3_)_6_:Sm^3+^ phosphor exhibits stable performance across a range of currents, making it a suitable candidate for application in w-LEDs.

## 3. Experimental Procedure

Red-emitting Sr_6_LuAl(BO_3_)_6_:*x*Sm^3+^ (1 ≤ *x* ≤ 30 mol%) phosphors were prepared via a high-temperature solid-state method. Lu_2_O_3_ (Aladdin.), Al_2_O_3_ (Macklin.), SrCO_3_ (Aladdin.), H_3_BO_3_ (Yonda Chemical), and Sm_2_O_3_ (Aladdin.) were employed as the starting reagents. The reaction was determined by employing the following equation:

$$\begin{array}{l} 6SrCO_{3}+0.5\left(1-x\right)Lu_{2}O_{3}+0.5xSm_{2}O_{3}+0.5Al_{2}O_{3}+\\ 6H_{3}BO_{3}\overset{1000{}^{\circ}C,\;4h}{\to }Sr_{6}Lu_{1-x}SmxAl{\left(BO_{3}\right)}_{6} \end{array}$$

The reagents were meticulously weighed according to the stoichiometric ratios above and ground in an agate mortar for 15 min to ensure homogeneity. The well-ground mixture was transferred to an alumina crucible and calcined in a muffle furnace at 1000 °C for 4 h in an air atmosphere. After the sintering process, the samples were allowed to return to an ordinary temperature by natural cooling and were then ground evenly for subsequent performance evaluations.

The phase purity of the synthesized powders was assessed using X-ray diffraction (XRD) analysis, employing a Rigaku Ultima IV diffractometer (Rigaku Corporation, Tokyo, Japan) with a Cu Kα X-ray source operated at 40 mA and 40 kV. The surface morphology testing and elemental mapping of Sr_6_LuAl(BO_3_)_6_:15 mol% Sm^3+^ were performed through a Hitachi SU5000 scanning electron microscope (SEM) (Hitachi, Tokyo, Japan) with an energy-dispersive spectrometer (EDS). The particle size distribution of the products was analyzed using a particle size analyzer (Malvern, MS2000, Malvern City, UK). The photoluminescence spectra were measured by an FLS-980 (Edinburgh) photoelectron spectrometer. The luminescence spectra at different temperatures from 300 to 480 K were obtained by an F-4600 (Hitachi, Tokyo, Japan) fluorescence spectrophotometer. The decay time and internal quantum efficiency (IQE) were measured by an FLS 1000 (Edinburgh, Livingston, UK) spectrometer equipped with a 450 W continued-wavelength xenon lamp, a pulsed xenon lamp, and an integrating sphere coated with BaSO_4_. The electroluminescence (EL) spectra of w-LEDs were investigated by an Ocean Optics’ USB 4000 fiber optic spectrometer (Ocean Optics, Florida, USA). The luminous efficacy was recorded via an integrating-sphere spectroradiometer system (HAAS2000, Everfine, Hangzhou, China).

## 4. Conclusions

This study reports the successful synthesis of a series of red-emitting Sr_6_LuAl(BO_3_)_6_:Sm^3+^ phosphors via a high-temperature solid-state reaction. The synthesized Sr_6_LuAl(BO_3_)_6_:Sm^3+^ samples, which were of pure phase and crystallized in the hexagonal system with an $R\bar{3}$ space group, exhibited a strong red emission centered at 599 nm when excited by near-ultraviolet light at 404 nm. The optimal doping concentration of Sm^3+^ ions was determined to be 10 mol%, at which nearest-neighbor ion interaction was identified as the primary mechanism for concentration quenching. The Sr_6_LuAl(BO_3_)_6_:Sm^3+^ phosphors demonstrated exceptional thermal stability, characterized by a high quenching temperature (*T*_0.5_ > 480 K) and a moderate activation energy (*E*_a_ = 0.24 eV). The IQE was measured as 26.3%. Furthermore, the color and color purity of these phosphors were found to be minimally influenced by variations in the Sm^3+^ ion doping concentration and temperature. The fabricated white light-emitting diode incorporating these phosphors had a favorable correlated color temperature of 5464 K, a high color rendering index of 95.6, and excellent CIE color coordinates of (0.333, 0.336). Collectively, these findings indicate that the Sr_6_LuAl(BO_3_)_6_:Sm^3+^ phosphors are well suited as red-emitting components for w-LED applications.

## Figures and Tables

**Table 1:** The refined structural data of Sr_6_LuAl(BO_3_)_6_:Sm^3+^ phosphors.

Formula	Sr_6_LuAl(BO_3_)_6_: 1 mol%Sm^3+^	Sr_6_LuAl(BO_3_)_6_: 10 mol%Sm^3+^
Space group	$R\bar{3}$	$R\bar{3}$
Cell parameters	*a* = *b* = 12.1565 Å, *c* = 9.0853 Å *α* = *β* = 90^°^, *γ* = 120^°^	*a* = *b* = 12.1736 Å, *c* = 9.0912 Å *α* = *β* = 90^°^, *γ* = 120^°^
Unit cell volume	*V* = 1162.7538 Å^3^	*V* = 1162.9426 Å^3^
*R*_wp_	7.5%	8.1%
*R*_p_	4.6%	5.3%
χ^2^	1.38	2.15

**Table 2:** The CIE chromaticity coordinates, CCT, and color purity of Sr_6_LuAl(BO_3_)_6_:Sm^3+^ samples.

Concentration (*x*/mol%)	Chromaticity Coordinates (*x*, *y*)	Color Purity (%)	CCT (K)
1	(0.589, 0.409)	99.6	1559
3	(0.594, 0.405)	99.9	1513
5	(0.595, 0.404)	99.9	1507
10	(0.595, 0.404)	99.9	1502
15	(0.594, 0.404)	99.6	1513
20	(0.594, 0.405)	99.9	1518
30	(0.591, 0.407)	99.6	1540

**Table 3:** The *T*_0.5_ quenching temperatures of Sm^3+^-activated phosphors.

Phosphors	*T*_0.5_ (K)	Ref.
Sr_6_LuAl(BO_3_)_6_:Sm^3+^	>480 K	This work
SrGd_2_O_4_:Sm^3+^	375 K	[36]
Li_6_SrLa_2_Nb_2_O_12_:Sm^3+^	462 K	[37]
Li_6_(Ca,Sr)La_2_Sb_2_O_12_:Sm^3+^	401 K	[38]
SrBi_4_Ti_4_O_15_:Sm^3+^	406 K	[39]
Gd_2_WTiO_8_:Er^3+^, Sm^3+^	493 K	[40]

**Table 4:** The CCTs, color rendering indices, and CIE coordinates of various phosphors.

Phosphor	CIE Coordinates	*R*_a_	CCT (K)	Ref.
Sr_6_LuAl(BO_3_)_6_:Sm^3+^	(0.333, 0.336)	95.6	5464	This work
CaY_2_Al_4_SiO_12_:Ce^3+^, Mn^2+^	(0.333, 0.327)	90.5	5460	[48]
Ba_6_Gd_2_Ti_4_O_17_:Eu^3+^	(0.390, 0.390)	82.2	3756	[49]
Sr_2_LaGaO_5_:Dy^3+^, Sm^3+^	(0.336, 0.314)	91.4	5284	[50]
Li_3_Ba_2_Gd_3_(WO_4_)_8_:Dy^3+^, Tm^3+^	(0.296, 0.355)	/	7106	[51]
CaGd_2_(MoO_4_)_4_:Sm^3+^	(0.305, 0.318)	82.6	7069	[52]
ScCaOBO_3_:Ce^3+^, Mn^2+^	(0.322, 0.332)	93.7	6014	[53]
Li_3_Y_3_BaSr(MoO_4_)_8_:Sm^3+^, Eu^3+^	(0.303, 0.368)	83	6645	[54]
Mg_2_YVO_6_:Eu^3+^	(0.369, 0.407)	89	4495	[55]

## Data Availability

The original contributions presented in the study are included in the article, further inquiries can be directed to the corresponding authors.
